# RNA interference (RNAi) screening approach identifies agents that enhance paclitaxel activity in breast cancer cells

**DOI:** 10.1186/bcr2595

**Published:** 2010-06-24

**Authors:** Joshua A Bauer, Fei Ye, Clayton B Marshall, Brian D Lehmann, Christopher S Pendleton, Yu Shyr, Carlos L Arteaga, Jennifer A Pietenpol

**Affiliations:** 1 Department of Biochemistry, Vanderbilt-Ingram Cancer Center, Vanderbilt University School of Medicine, Preston Research Building, 2200 Pierce Avenue, Nashville, TN 37232, USA; 2 Department of Biostatistics, Vanderbilt-Ingram Cancer Center, Vanderbilt University School of Medicine, Preston Research Building, 2200 Pierce Avenue, Nashville, TN 37232, USA; 3 Department of Medicine, Vanderbilt-Ingram Cancer Center, Vanderbilt University School of Medicine, Preston Research Building, 2200 Pierce Avenue, Nashville, TN 37232, USA

## Abstract

**Introduction:**

Paclitaxel is a widely used drug in the treatment of patients with locally advanced and metastatic breast cancer. However, only a small portion of patients have a complete response to paclitaxel-based chemotherapy, and many patients are resistant. Strategies that increase sensitivity and limit resistance to paclitaxel would be of clinical use, especially for patients with triple-negative breast cancer (TNBC).

**Methods:**

We generated a gene set from overlay of the druggable genome and a collection of genomically deregulated gene transcripts in breast cancer. We used loss-of-function RNA interference (RNAi) to identify gene products in this set that, when targeted, increase paclitaxel sensitivity. Pharmacological agents that targeted the top scoring hits/genes from our RNAi screens were used in combination with paclitaxel, and the effects on the growth of various breast cancer cell lines were determined.

**Results:**

RNAi screens performed herein were validated by identification of genes in pathways that, when previously targeted, enhanced paclitaxel sensitivity in the pre-clinical and clinical settings. When chemical inhibitors, CCT007093 and mithramycin, against two top hits in our screen, PPMID and SP1, respectively, were used in combination with paclitaxel, we observed synergistic growth inhibition in both 2D and 3D breast cancer cell cultures. The transforming growth factor beta (TGFβ) receptor inhibitor, LY2109761, that targets the signaling pathway of another top scoring hit, TGFβ1, was synergistic with paclitaxel when used in combination on select breast cancer cell lines grown in 3D culture. We also determined the relative paclitaxel sensitivity of 22 TNBC cell lines and identified 18 drug-sensitive and four drug-resistant cell lines. Of significance, we found that both CCT007093 and mithramycin, when used in combination with paclitaxel, resulted in synergistic inhibition of the four paclitaxel-resistant TNBC cell lines.

**Conclusions:**

RNAi screening can identify druggable targets and novel drug combinations that can sensitize breast cancer cells to paclitaxel. This genomic-based approach can be applied to a multitude of tumor-derived cell lines and drug treatments to generate requisite pre-clinical data for new drug combination therapies to pursue in clinical investigations.

## Introduction

Chemotherapy regimens containing taxanes, including docetaxel and paclitaxel, have well-established benefits in breast cancer [1,2]. Despite improvement in the response rates with use of taxane-based drug combinations versus single agent taxanes, most patients do not have a complete response to treatment [3-6]. A partial response or resistance to paclitaxel is a major limiting factor in the successful treatment of breast cancer. Improving taxane-based chemotherapy regimens through novel drug combinations is therefore of clinical interest. Patients with tumors that lack expression of estrogen receptor (ER), progesterone receptor (PR), and HER2 amplification (triple-negative breast cancer, TNBC) are not candidates for currently available FDA-approved, targeted therapies. More efficacious combination chemotherapy is needed for these patients.

Due to its extensive use in breast cancer and other tumor types and the frequency of acquired resistance, mechanisms of taxane resistance have been investigated [7-9]. Some mechanisms identified to date include mutations of the β-tubulin gene [10,11], expression of the tubulin binding protein tau [12], expression of ER [13,14], HER2 [15,16], BRCA1 [17,18], and p-glycoprotein/MDR1 [19-21], among others [8,9]. Genomic studies have also been used for predicting response to both paclitaxel and related compound docetaxel [3,5,6,22,23], but few if any genes amongst these studies overlap or have been confirmed as reliable markers or predictors of response. Despite these studies, novel therapeutic combinations with paclitaxel are being tested in clinical trials, especially in patients with advanced disease or those without clinically proven therapeutic targets such as TNBC [24-26]. Identification of gene products that when pharmacologically inhibited enhance paclitaxel sensitivity may lead to improved response rates and reduced resistance.

The advent of RNA interference (RNAi) for gene silencing allows for systematic gene and/or pathway analysis in tumor cells and an ability to uncover novel gene functions and pathways that cannot always be identified by ectopic gene expression. Several RNAi studies performed in human tumor cell lines using synthetic small interfering RNAs (siRNAs) or vector-based short hairpin RNAs (shRNAs) targeting defined gene families or genome-wide libraries have identified modulators of drug sensitivity [27-33]. These studies have unveiled novel pathways and molecules for therapeutic targeting in various tumor types and there is a great need to translate this information for clinical utility.

Genomic tumor profiling has provided us with important insights to mechanisms of tumorigenesis and translational data for clinical advances. Relative to some cancer types, there is tremendous genomic information available for breast cancers, which includes tumor DNA copy number [34-38], DNA sequence and mutations [39-44], gene expression and protein profiles [45,46], as well as epigenetics [47,48] and microRNAs [49,50]. In the current study, we performed genetic loss-of-function RNAi screens to identify druggable targets involved in paclitaxel sensitivity. In our screens, we used a gene set that is comprised of the overlay of a druggable genome library with a set of genes considered to be deregulated in breast cancer (from genomic studies of human breast cancers and cell lines [37,38]). Specific pharmacological inhibitors of the top scoring hits from our screens were used in combination with paclitaxel and the ability of the chemicals to enhance the growth inhibitory activity of paclitaxel on breast tumor-derived cell lines was analyzed. We further tested these novel paclitaxel drug combinations on four paclitaxel-resistant TNBC cell lines and for select inhibitors showed synergistic drug activity. New findings presented in this study show the feasibility of loss-of-function screening to provide biological relevance for genomic discoveries and to identify drug combinations to improve current taxane-based drug treatments in pre-clinical models for breast cancer.

## Materials and methods

### Reagents and resources

Paclitaxel, CCT007093, and mithramycin A (Sigma-Aldrich, St. Louis, MO, USA) were prepared in DMSO at a stock concentration of 0.1 mM, 5 mM, and 0.9 mM, respectively. LY2109761 was kindly provided by Jonathan Yingling, Lilly Research Laboratories, Indianapolis, IN, USA and prepared in DMSO at 10 mM stock concentration. The panel of candidate genes used in the shRNA screen was generated from overlay of a list of 1,778 genomically deregulated gene transcripts whose levels significantly correlated with genome copy number in breast cancer [37,38] and a druggable genome list compiled from two sources (Open Biosystems, Huntsville, AL, USA and Qiagen, Valencia, CA, USA). Pharmacological agents were identified using several drug databases including DrugBank, Therapeutic Target Database, Comparative Toxicogenomics Database, and Ingenuity Pathway Analysis.

### Cell culture

HeLa and MCF-7 cells were purchased from American Tissue Cell Culture (ATCC, Manassas, VA, USA) and cultured in Dulbecco's modified Eagle's medium (DMEM, Invitrogen, Carlsbad, CA, USA) supplemented with 10% fetal bovine serum, and 1% penicillin-streptomycin. All TNBC cell lines were purchased from ATCC or Deutsche Sammlung von Mikroorganismen und Zellkulturen GmbH (DSMZ, Braunschweig, Germany) and cultured as described (Additional File 1). All cells were cultured at 37°C with 5% CO_2 _and tested routinely for mycoplasma, using the MycoAlert Detection Kit (Cambrex, Rockland, ME, USA).

### shRNA and siRNA screens

HeLa cells were plated at 20,000 cells per well (96-well plate) and 24 h later transfected with a subset of the human genome pGIPZ shRNAmir plasmid library (n = 1,078) (Open Biosystems), as provided by the Functional Genomics Shared Resource at Vanderbilt University in a one clone per well format. The next day, cells were split 1:6 into 96-well plates, allowed to attach overnight, and three plates were treated with vehicle control (DMSO) and three were treated with 5 nM paclitaxel for 24 h. Cells were washed, replaced with fresh media and incubated for an additional 72 to 96 h. Alamar Blue (Invitrogen), a dye used to detect metabolic activity in cells, was used to assay for cell viability and to identify genes that alter paclitaxel sensitivity. To identify gene targets that promote paclitaxel sensitivity or resistance, we generated a sensitivity index (SI) score for each shRNA obtained from replicate experiments after drug treatment [32]. The SI score accounts for both the individual effect of shRNAs and the effect of drug on cell viability (see next section for description of the statistical methodology). Data from each plate were normalized to non-silencing (NS) shRNA controls that do not target any human gene, to account for plate-to-plate variability and to control for the effects of shRNA transfection. For the siRNA screen, two independent siRNAs were designed for each gene and randomly distributed in a 96-well plate. MDA-MB-231 and MDA-MB-468 cells were reverse-transfected with siRNAs complexed with lipid reagent for 48 h and subsequently split into four replicate plates. Cells were treated and measured for viability in a similar fashion as above. Transfections (that is, experiments) were performed in triplicate to allow for assessment of variation of expression data in statistical analysis.

### Statistical analysis

Median centered global normalization was performed across all shRNA and siRNA plates by using the NS controls in each plate. The SI score was calculated for each of the shRNAs and siRNAs by estimating the difference between the expected and observed combined effects of shRNAs or siRNAs and paclitaxel on cell viability, as previously described [32]. The SI scores range from -1 to 1. Positive SI scores indicate sensitizing effects and negative SI scores indicate antagonizing effects.

A bootstrap algorithm was used to estimate the variability of the mean SI level for each gene with > 3 shRNAs by randomly sampled from all shRNAs of that gene with replacement. The corresponding 95% percentile bootstrap confidence interval was calculated for each gene. Genes were taken as hits if they had a mean bootstrap in the upper quartile cutoff SI > 0.078 and the lower bound of 95% confidence interval > 0. The results of a small simulation study we carried out show that the bootstrap distribution from a very small number of shRNAs (≤3 per gene) is not reliable. Therefore, the mean SI value was calculated for the genes with ≤3 shRNAs. A more stringent cutoff (SI > 0.15) was used for hit selection among these genes. For the siRNA screen, the SI value was calculated by averaging the two siRNAs for each gene after normalization and the top hits for each cell line were selected based on the SI value of the averaged data. Correlation between experiments was estimated using Pearson's correlation coefficient. Statistical analysis was performed using R software (version 2.10.1).

### Cell growth and viability assays

For cell growth assays cells were seeded at 5 × 10^5 ^cells per well of a six-well plate. The next day cells were treated with 5 μM CCT007093 or 10 nM mithramycin, ± 3 nM paclitaxel, or vehicle control (DMSO). After three days cells were collected, washed, and counted using a Coulter Counter (Beckman-Coulter, Brea, CA, USA). Cell number was plotted as a percent of cells relative to vehicle control. Cell viability assays were performed by seeding 3,000 to 8,000 cells per well of a 96-well plate. The next day, growth media was replaced with treatment media containing vehicle-DMSO or paclitaxel that was serial diluted by half-log concentrations ranging from 0.3 to 30 nM. After three days of incubation with the drug, cell viability was measured using the Alamar Blue assay (Invitrogen). Cell viability for each drug concentration was compared to vehicle-treated control. Four replicate wells from three independent experiments of each drug concentration were used to generate median-effect plots to calculate the IC_50 _(concentration for 50% growth inhibition) concentrations for each cell line using Calcusyn Software (Biosoft, Cambridge, United Kingdom). IC_50 _values for each cell line are represented with standard error.

### Mammosphere cultures

For three-dimensional (3D) mammosphere cultures, cells were seeded on growth factor-reduced Matrigel (BD Biosciences, San Jose, CA, USA) in chamber slides as previously described [51,52]. CCT007093, mithramycin, and LY2109761 ± paclitaxel were added to medium 24 h after cell seeding and medium was replaced every three days. Mammospheres were detached from Matrigel with dispase enzyme (BD Biosciences), trypsinized into single cell suspensions, and cell number was determined using a hemocytometer. The number of viable cells was plotted as a percent of cells relative to vehicle control.

### Drug synergy analysis

Paclitaxel was combined with each of the different agents at a fixed ratio (1:1) of the individual IC_50 _concentrations of each drug. Drug combinations were then serial diluted (1:2) and represented as IC_50_, IC_25_, and IC_12.5 _concentrations, as the additive effects of both drugs. Statistical analysis of drug synergy was evaluated from the results of the Alamar Blue assays and calculated using the Chou-Talaly method [53] and Calcusyn Software (Biosoft). To determine synergy between two drugs, the software uses a median-effect method that determines if the drug combination produces greater effects together than expected from the summation of their individual effects. The combination index (CI) values are calculated for the different dose-effect plots (for each of the serial dilutions) based on the parameters derived from the median-effect plots of the individual drugs or drug combinations at the fixed ratios. The CI was calculated based on the assumption of mutually nonexclusive drug interactions. CI values significantly > 1 are antagonistic, not significantly different than 1 are additive, and values < 1 are synergistic. Two-sided statistical tests were used to determine if the mean CI values resulting from three independent experiments at multiple effect levels were statistically significantly different from a CI = 1.

## Results

### RNAi screening for genes that sensitize cells to paclitaxel

**Figure 1:**
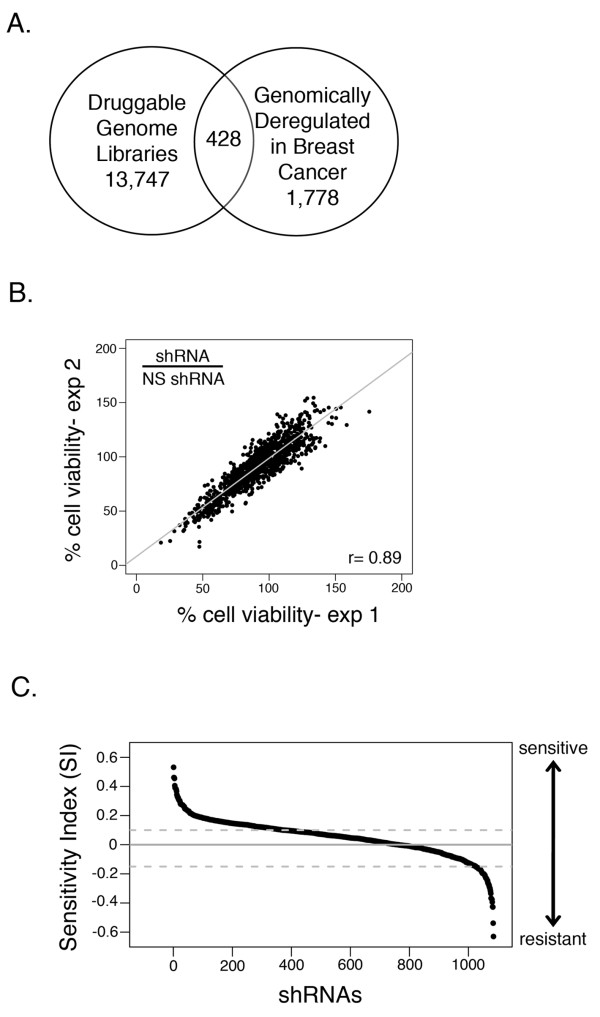
**shRNA screen to identify paclitaxel sensitizers**. **A**. The overlay of druggable genome libraries (Qiagen and Open Biosystems) and genes deregulated in breast cancer resulted in 428 candidate druggable genes. **B**. Reproducibility of shRNA screen by correlation of the effect of shRNAs on cell growth compared to non-silencing shRNA in vehicle-treated plates of two replicate experiments. Spearman correlation coefficient, r = 0.89. **C**. Each shRNA was scored for the level of paclitaxel sensitivity using the sensitivity index (SI) as described in Materials and Methods. The SI score ranges from -1 to 1. Positive significant SI scores indicate sensitization and negative significant SI scores indicate antagonism. The scatter plot of all shRNAs is shown in rank order. The dashed lines indicate the relative threshold of significant drug sensitivity.

To identify druggable gene targets that could enhance paclitaxel activity in breast cancer cells, we performed an shRNA screen. We selected a subset of genes based on a comprehensive genomic study of 145 primary human breast tumors and 51 breast cancer cell lines in which 1,778 gene transcripts were identified whose levels significantly correlated with genome copy number and are deemed genomically deregulated in breast cancer [37]. Most of the alterations present in primary tumors were retained in the cell lines [37]. The 1,778 genomically deregulated genes were overlaid with a druggable gene list (compiled from two sources, Open Biosystems and Qiagen), with the expectation that for select genes identified in the shRNA screen, an agent may already exist that could be analyzed in preclinical models for synergistic activity with paclitaxel. The overlay of the gene lists yielded 428 genes (Figure 1A). From a whole-genome vector-based shRNAmir library, we generated a sub-library consisting of 1,078 shRNAs targeting the 428 genes, with 1 to 11 shRNAs per gene. Since the transfection efficiency of plasmid-based vectors in most breast cancer cell lines is < 10%, we used a highly transfectable cell line, HeLa, for our primary screen with the assumption that genes/pathways related to paclitaxel sensitivity are conserved across cancer cell lines. Positive hits from the first screen in HeLa cells were validated in secondary screens using two triple-negative breast cancer (TNBC) cell lines as described below.

shRNAs for each gene in our sub-library were independently transfected into HeLa cells in a 96-well-plate format and cells were split 24 h after transfection into six replicate plates. After 48 h, half of the plates (n = 3) received an IC_50 _concentration of paclitaxel (5 nM) and half received vehicle (DMSO) treatment. In order to detect significant differences in drug sensitivity in the assay, we allowed time for multiple cell divisions. After four days of drug treatment, cell viability was measured using an Alamar Blue assay to identify genes that alter paclitaxel sensitivity (effect of shRNA and drug). Comparison of the mean viability values of three replicates for each shRNA from the two individual screens revealed high reproducibility (r = 0.89, Pearson's correlation coefficient) (Figure 1B). We combined the results from the duplicate screens in the final analyses.

To account for plate-to-plate variability, we normalized across all the plates using non-silencing (NS) control shRNAs that were present in each plate. To identify genes that when targeted promote paclitaxel sensitivity or resistance; we generated a sensitivity index (SI) score for each shRNA obtained from replicate experiments after drug treatment, as previously described (Figure 1C) [32]. The SI score accounts for the individual effect of shRNAs and the effect of drug on cell viability. A positive SI score is a measure of sensitivity and a negative SI score is indicative of resistance to paclitaxel treatment. In this study, we chose gene targets that are amplified/overexpressed in breast and that increase paclitaxel sensitivity (+SI value), as these are more likely to be better targets for pharmacological inhibition.

For selection of hits from our primary shRNA screen, we used a bootstrap algorithm to identify gene targets that had > 3 shRNAs based on the mean SI > 0.078 (upper quartile) and the corresponding 95% confidence interval (Table 1). These criteria allowed for high-confidence hits to be selected. As the number of positive scoring (+SI) shRNAs for each gene increased, our confidence for these genes increased, as these are unlikely due to false-positives or off-target effects of individual shRNAs. However, since this method biased our hit selection for those genes that had more shRNAs in our sub-library, we selected additional hits represented by genes that had ≤3 shRNAs but with a much more stringent cutoff of mean SI value > 0.150 (Table 1). FRAP1 (mTOR) (mean SI = 0.212; Table 1) was previously identified through an RNAi screen as a target of paclitaxel sensitivity, and was used in our screen as a positive control in each plate [30]. CASP3 shRNA (mean SI = -0.042) was used as a negative control in each plate as we found that this gene, when downregulated, induces paclitaxel resistance (Table 1). Three of the four shRNAs that target EGFR were highly sensitive to paclitaxel activity (mean SI = 0.136, Table 1). EGFR is a known target of paclitaxel sensitivity as erlotinib, an EGFR inhibitor, increases paclitaxel activity *in vivo *[54-57]. Additionally, TUBG1, tubulin gamma-1, a component of the γ-tubulin ring complex (γ-TuRC), involved in mitotic spindle formation, enhanced paclitaxel sensitivity (mean SI value = 0.152, Table 1). γ-TuRC has previously been shown to enhance paclitaxel sensitivity, *in vitro *[33]. These data collectively validated our primary shRNA screening approach.

**Table 1:** Paclitaxel sensitivity index for indicated genes from shRNA screen

	> 3 clones mean SI > 0.078					< 3 clones mean SI > 0.150		
Gene	shRNAs	Mean SI	95% CI	Genomic dereg*	Gene	shRNAs	Mean SI	Genomic dereg*
YWHAZ	6	0.193	0.154 to 0.242	amp/OE	PCK1	1	0.461	amp/OE
RPS6KB1	4	0.186	0.135 to 0.242	amp/OE	SREBF2	1	0.391	amp/OE
COG2	5	0.186	0.110 to 0.265	amp/OE	SRC	1	0.276	amp/OE
PTK2	5	0.184	0.121 to 0.287	amp/OE	FNTA	1	0.263	amp/OE
PPM1D	4	0.179	0.120 to 0.241	amp/OE	BCL2L1	1	0.247	amp/OE
SKP1A	6	0.166	0.075 to 0.259	amp/OE	COMMD1	3	0.220	amp/OE
MARK1	5	0.157	0.006 to 0.345	amp/OE	COG8	1	0.214	amp/OE
NFYB	4	0.148	0.085 to 0.186	amp/OE	FRAP1	1	0.212	NA
RBBP4	4	0.139	0.080 to 0.198	amp/OE	ERBB2	1	0.180	amp/OE
IL10	4	0.136	0.081 to 0.205	amp/OE	IQGAP1	3	0.176	amp/OE
EGFR	4	0.136	0.087 to 0.378	amp/OE	PHB	3	0.159	amp/OE
SP1	5	0.130	0.080 to 0.138	amp/OE	NDUFS6	1	0.159	amp/OE
STX16	4	0.107	0.053 to 0.175	amp/OE	COG1	1	0.159	amp/OE
PTPN7	4	0.095	0.019 to 0.170	amp/OE	PRPF4B	3	0.156	amp/OE
SENP1	5	0.095	0.022 to 0.168	amp/OE	FADD	3	0.156	amp/OE
CENPF	4	0.085	0.030 to 0.140	amp/OE	ERK1	1	0.154	amp/OE
IGF1	4	0.078	0.016 to 0.137	amp/OE	TGFB1	1	0.153	amp/OE
					TUBG1	3	0.152	amp/OE
CASP3 (control)	5	-0.042	-0.061 to -0.021	del/UE	IKBKB	2	0.151	amp/OE

*as determined by DNA copy number and gene expression analysis by Neve *et al*.

SI, sensitivity index; CI, confidence interval

amp/OE, amplification/overexpressed; del/UE, deletion/underexpressed

NA, not available

To determine if the results of the shRNA screen were reproducible in breast cancer cells, we validated the top 36 high-confidence hits (genes) from the shRNA screen that were amplified/overexpressed in breast cancer and had positive SI values (Table 1). Some of the genes selected are targets of agents that have not been tested for efficacy in combination with paclitaxel in the preclinical setting and are of biological relevance and interest (for example, transforming growth factor beta (TGFβ) signaling). Two independent siRNA oligos were designed for each of the 36 genes selected and reverse-transfected into two TNBC cell lines, MDA-MB-231 and MDA-MB-468. Duplicate experiments were performed and resulted in high reproducibility (correlation coefficients approximately 0.70 to 0.80, data not shown).

We averaged the SI value for the two siRNAs from duplicate experiments for each gene and the top hits for each cell line were selected for further analysis (Table 2). Four genes, PPM1D, CENPF, BCL2L1, and FRAP1 were sensitizers of paclitaxel in both cell lines (bold, Table 2). Since paclitaxel efficacy is dependent on mitotic activity (that is, cell cycle transit into M-phase), we postulated that siRNAs that decreased cell viability > 30% in untreated plates were unlikely candidates for enhancing paclitaxel activity as cell cycle slowing or arrest limits the efficacy of paclitaxel. However, we did note the effect that some siRNAs had on breast cancer cell viability in untreated plates as the targeted gene may be of potential interest for further investigation for breast cancers that do not have targeted therapy, such as TNBC. For example, IGF1 siRNA in MDA-MB-468 cells led to a 60% reduction in viability compared to NS siRNA control (data not shown). However, we did not observe significant sensitivity to paclitaxel (SI = -0.031) for IGF1 siRNAs in these cells, likely due to the large loss of cell viability prior to paclitaxel treatment.

**Table 2:** Top gene targets from siRNA screen that increase paclitaxel sensitivity and the corresponding chemical inhibitors

	MDA-MB-231	MDA-MB-468		Previous combination with paclitaxel		
				
Gene	Mean SI	Mean SI	Drug/chemical inhibitor	Pre- clinical	Clinical	Ref
PPM1D	0.055	0.136	thioxanthen-9-one; CCT007093; anti-estrogens*	no	no	
CENPF	0.049	0.113	farnesyltransferase inhibitors*	yes	yes	[62,68,69]
BCL2L1	0.041	0.093	ABT-737; AT-101	yes	no	[63,64]
FRAP1	0.037	0.078	rapamycin; RAD001	yes	yes	[58,59]
IGF1	0.038		NVP-AEW541; 9-cis-retinoic acid*; raloxifene*	yes	no	[67]
EGFR		0.154	erlotinib; gefitinib; cetuximab	yes	yes	[54,60,61]
ERK1		0.148	ERK/MEK inhibitors	yes	no	[65,66]
RPS6KB1		0.140	rapamycin*; RAD001*	yes	yes	[58,59]
TGFB1		0.121	LY2109761; LY2157299; SD-208	no	no	
SP1		0.085	mithramycin; arsenic trioxide*	no	no	

*indirect inhibitors

SI, sensitivity index

To ensure that drug sensitivity correlated with relative decreases in gene expression and to eliminate any possible off-target effects from shRNAs and siRNAs, we used Dharmacon ON-TARGET*plus *individual and pooled siRNAs as a third independent RNAi approach on select positive hits and our results with PPMID are shown as an example. ON-TARGET*plus *siRNAs for a top hit, PPM1D, were transfected in two breast cancer cell lines, MCF-7 and MDA-MB-468. PPM1D knockdown was measured at 48 h after transfection by quantitative real-time PCR. Three of the four individual and the pooled ON-TARGET*plus *siRNAs for PPM1D showed > 80% reduction in PPM1D mRNA levels in MCF-7 cells and > 60% reduction in MDA-MB-468 cells (Additional File 2). Importantly, knockdown of PPM1D was correlated with increased paclitaxel sensitivity over a range of paclitaxel doses in both cell lines (Additional File 2). The use of multiple shRNAs and validation with independent siRNAs limited the likelihood that the observed sensitivity was due to off-target effects.

### Candidate pharmacological inhibitors that enhance paclitaxel sensitivity

A primary goal of this study was to identify gene targets that are druggable, to which pharmacological agents have been developed, and that can be used in novel combinations with paclitaxel in preclinical studies. The list of top hits from the validation siRNA screen for both cell lines is shown in Table 2 with associated chemical agents identified using *in silico *drug databases (see Materials and Methods). In some cases, agents linked to genes in the list represent FDA-approved drugs, some of which have already been successfully used in combination with paclitaxel (for example, FRAP1; rapamycin [58,59], EGFR; erlotinib [54,60,61]). Gene targets with inhibitors known to enhance paclitaxel sensitivity both in preclinical [62-67] and clinical models [54,58,61,68,69] (noted in Table 2) were not studied further; however, their discovery validated our RNAi screening approach. We also did not pursue hits that had non-specific inhibitors and those that had no available agents despite being considered druggable (for example, MARK1); however, those gene targets still remain of interest. Since some hits are involved in intricate signaling pathways, there could be other drug targetable molecules within the same pathway, which could impact paclitaxel sensitivity. For example, a top hit in our screen, RPS6KB1, is downstream of mTOR and PI3K, two prominent signaling pathways in breast cancer with known direct inhibitors, rapamycin and LY294002, and that have been shown to sensitize cells to paclitaxel [59,70].

Three gene targets from our list were of particular interest. These genes encode proteins to which agents have been developed and thus we could test the compounds in combination with paclitaxel for biological effect. The first was PPM1D, a member of the PP2C family of serine/theronine protein phosphatases, and a known negative regulator of cell stress response pathways including those regulated by p53, CHEK1, and p38 MAP kinase [71]. PPM1D is amplified and overexpressed in breast cancers [72,73] and inhibition of its activity, through use of small molecules such as CCT007093, inhibits the growth of tumor cell lines that overexpress PPM1D [74,75]. The second gene target of interest was SP1, a constitutively expressed transcription factor that regulates basal promoter activity of many housekeeping genes. SP1-binding activity has been shown to be higher in human breast carcinomas than in normal tissues and may play a role in tumorigenesis by regulating the expression of genes involved in angiogenesis, cell growth, and apoptosis resistance [76,77]. Mithramycin A binds to dsDNA and inhibits SP1 binding sites (GC-rich regions of promoters) thus inhibiting SP1 transcriptional activity [78]. Finally, TGFβ1 is a ligand that regulates a signaling pathway that becomes deregulated in many types of malignancies including breast cancer [79]. TGFβ1 can act in a paracrine manner to promote tumor growth and can activate PI3K/AKT, a signaling program associated with drug resistance [80]. Thus, the ligand TGFβ1 and its receptors TGFβ receptor (TGFβR) type I and II have been pursued as anti-cancer targets. LY2109761 is a small molecule inhibitor of TGFβR I and II and has been shown to inhibit tumor cell migration, invasion, as well as suppressing metastasis *in vivo *[80-82].

### Pharmacological agents enhance paclitaxel cell growth inhibition of breast cancer cells

**Figure 2:**
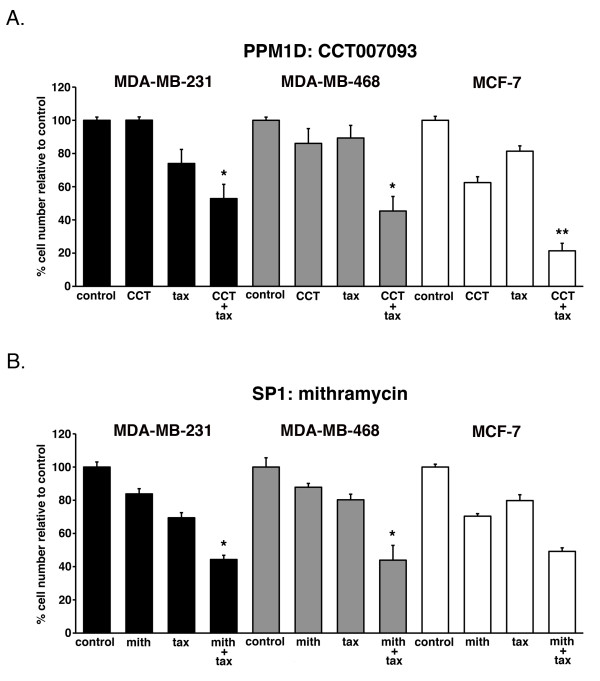
**Novel drug combinations sensitize breast cancer cells to paclitaxel**. **A**. MDA-MB-231, MDA-MB-468, and MCF-7 breast cancer cell lines were seeded in six-well plates and treated with vehicle (control), < IC_50 _concentrations of the putative PPM1D inhibitor, CCT007093 (CCT); paclitaxel (tax); or a combination of both (CCT + tax). Cells were treated for 72 h, washed, trypsinized and counted. The percent of viable cells relative to control was plotted for each drug or combination. **B**. Same as A with < IC_50 _concentration the putative SP1-binding inhibitor, mithramycin (mith). Error bars represent standard deviation of triplicates from three independent experiments. * indicates *P *< 0.05, ** indicates *P *< 0.01.

To observe potential enhanced activity of drug combinations, < IC_50 _concentrations of CCT007093 or mithramycin were combined with a < IC_50 _concentration of paclitaxel. These combinations resulted in increased growth inhibition of three breast cancer cell lines tested, MDA-MB-231, MDA-MB-468, and MCF-7 relative to single agent treatment (Figure 2A). CCT007093 alone had little effect on MDA-MB-231 or MDA-MB-468 cell growth (< 15% inhibition) but significantly decreased proliferation in combination with paclitaxel, 47% and 55% inhibition (*P < 0.05*), respectively. MCF-7 cells, which contain an amplification of PPM1D [73], are sensitive to single agent CCT007093 treatment (38% inhibition of cell growth, Figure 2A) and synergized with paclitaxel resulting in a 79% inhibition of cell growth (*P < 0.01*). Mithramycin in combination with paclitaxel also significantly inhibited cell growth in the triple-negative MDA-MB-231 and MDA-MB-468 cells relative to the effect observed when either drug was used alone (*P < 0.05*). However, mithramycin treatment of MCF-7 cells failed to enhance paclitaxel activity greater than the additive effects of either drug alone (additive effective = 50%, observed effect = 51%). Of note, we did not observe any appreciable drug effects on cell viability in 2D cultures with the TGFβR inhibitor LY2109761, alone or in combination with paclitaxel in parallel assays with the cell lines described above (data not shown).

### Novel drug combinations with paclitaxel inhibit 3D growth of breast cancer cell lines

**Figure 3:**
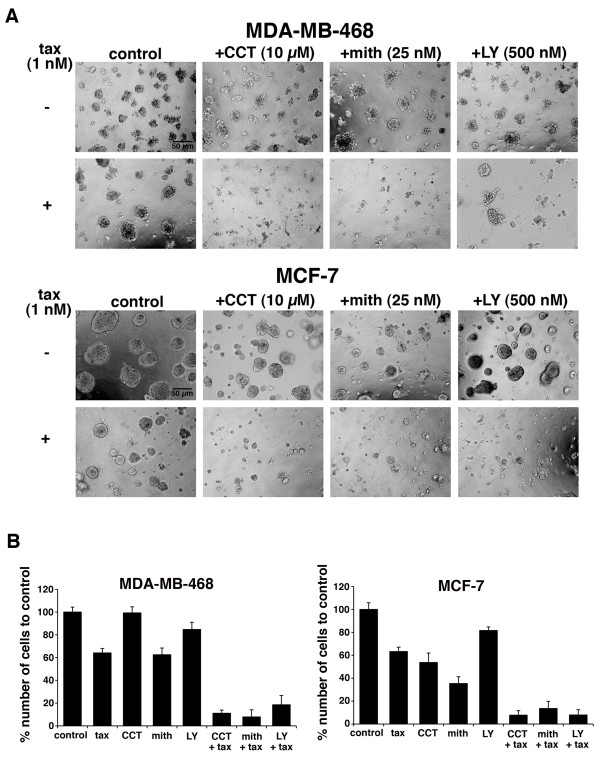
**Analysis of drug combinations on growth of breast cancer cells grown in 3D cultures**. **A**. Cells were seeded on Matrigel in eight-well chamber slides as described in Materials and Methods. 3D cultures formed after two days and were treated every two to three days with single agents, vehicle (control), 1 nM paclitaxel (tax), 500 nM LY2109761 (LY), 10 μM CCT007093 (CCT), 25 nM mithramycin (mith) (upper panels) or a combination of drugs (lower panels). After 10 to 14 days, mammospheres were visualized using phase-contrast microscopy. Bar scale, 50 μm. **B**. To count cell numbers, the Matrigel was dissolved, mammospheres were collected, trypsinized and single cells were counted by trypan blue exclusion assay using a hemocytometer. The percent cell number relative to control was plotted for each drug or combination for the two cell lines. Error bars represent standard deviation from replicates from three independent experiments.

To determine the effect of the novel drug combinations on paclitaxel sensitivity in 3D cultures, we grew two cell lines, MDA-MB-468 and MCF-7, as mammospheres, a culture method that has been developed to analyze breast epithelial function, morphology, and invasiveness [51,52]. Paclitaxel treatment alone reduced mammosphere formation and overall cell number by 37% in MCF-7 and 36% in MDA-MB-468 cells (Figure 3A, B). CCT007093 treatment alone reduced MCF-7 mammospheres by 46% versus < 1% reduction of MDA-MB-468 mammospheres, a line that does not have appreciable PPM1D expression levels. However, CCT007093 treatment enhanced paclitaxel sensitivity and reduced mammosphere cell number by 89% in MDA-MB-468 and 92% in MCF-7 cultures. Likewise, we observed a significant reduction in the number of cells in the mammospheres that formed with the combination of mithramycin and paclitaxel in both MDA-MB-468 and MCF-7 cells (92% and 86% reduction, respectively). Although we did not observe any appreciable drug synergy with the TGFβR inhibitor LY2109761 in 2D, monolayer cell culture, we did observe a significant effect in 3D cultures. When used in combination with paclitaxel, LY2109761 inhibited mammosphere formation and reduced cell number by 72% and 92% compared to control in MDA-MB-468 and MCF-7 cells, respectively; however, it had minimal effect on mammosphere cell growth when used as a single agent (< 20% reduction).

### CCT007093 and mithramycin synergistically enhance paclitaxel activity in paclitaxel-sensitive and -resistance TNBC cell lines

There are currently no targeted therapies for patients with TNBC. Frequently, patients with this type of breast cancer receive paclitaxel, due to its initial effects and higher response rates as compared to other chemotherapies [83]. However, not all patients have a complete response and those that are resistant or have residual disease after initial or secondary chemotherapy have a worse prognosis and outcome [83,84]. In addition, TNBC patients that initially respond to chemotherapy have a higher incidence and faster relapse compared to patients with non-TNBC [85]. Thus, improving the effect of initial paclitaxel treatment is an important goal in successfully treating patients with TNBC until more improved and/or targeted therapies are developed.

**Figure 4:**
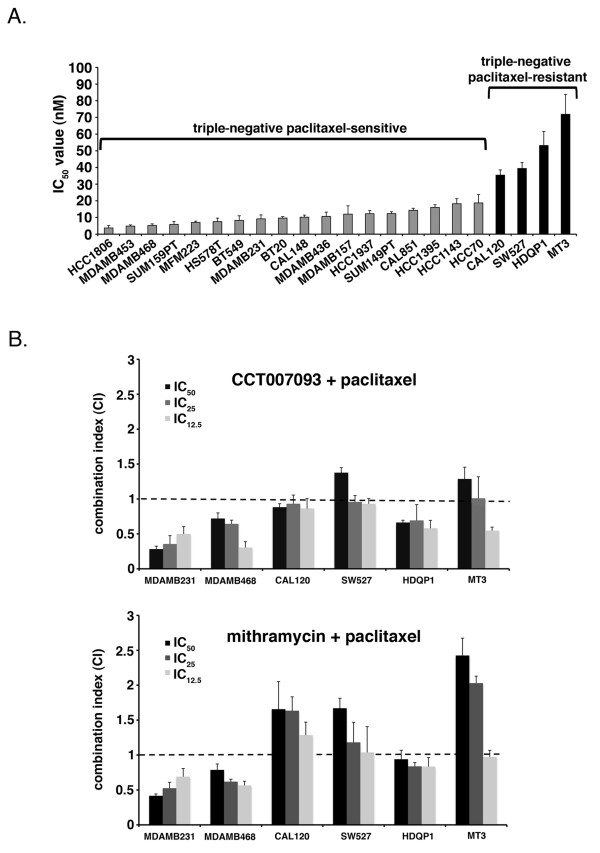
**Drug combinations to enhance cell death of TNBC cell lines**. A. Twenty-two triple-negative cell lines were each seeded in 96-well plates. The next day cells were treated with vehicle control or paclitaxel (0.3 to 30 nM). IC_50 _values for each cell line were generated based on the median-effect plot from three independent experiments. IC_50 _values represent the inhibitor concentration required for a 50% reduction in cell viability relative to vehicle-treated controls. Error bars represent standard deviation of four replicates from three independent experiments. **B**. Cell lines were seeded in 96-well plates and treated with single agents (IC_50 _values) or a combination of drugs (CCT007093 + paclitaxel or mithramycin + paclitaxel) of the IC_50 _concentrations of each drug (1:1 ratio) serial-diluted (IC_50_-IC_25_-IC_12.5_). Combination index (CI) values were calculated using the Chou-Talalay method with CalcuSyn software (Biosoft). CI values significantly > 1 are antagonistic, not significantly different than 1 are additive, and values < 1 are synergistic. Error bars represent standard deviation of quadruplicates from three independent experiments.

Along these lines, we determined the relative paclitaxel sensitivity of a panel of TNBC cell lines by determining the paclitaxel IC_50 _values for 22 TNBC cell lines (Figure 4A). The distribution of IC_50 _values across the panel led us to classify 18 cell lines as relatively paclitaxel-sensitive and four cell lines (CAL120, SW527, HDQP1, and MT3, which had relatively high IC_50 _values (> 20 nM)) as relatively paclitaxel-resistant. We determined if the four resistant cell lines could be sensitized to paclitaxel using the novel drug combinations presented above and assayed the two lines used in our RNAi screening, MDA-MB-231 and MDA-MB-468 for comparison (Figure 4A). A four-day cell viability assay after combination treatments was used to assess drug synergy, defined as the combination of two agents that have a greater therapeutic effect than would be expected by the addition of individual effects of each drug. The well-established Chou and Talalay method was used to determine drug synergy, as described in Materials and Methods [53]. Combination index (CI) values were derived from the median-effect plots of single agents alone or in combination and statistical tests were used to determine whether the CI values at multiple dose-effect levels (IC_50_, IC_25_, IC_12.5_) were statistically significantly different from 1 (*P < 0.05*). CI values significantly < 1 indicate synergy, not significantly different from 1 indicates additive, and a CI value significantly > 1 indicates antagonism.

CCT007093 was synergistic with paclitaxel (average CI value significantly < 1, *P < 0.05*) in two paclitaxel-sensitive cell lines, MDA-MB-468 and MDA-MB-231, average CI value of 0.56 and 0.38, respectively, and in two of the four paclitaxel-resistant cell lines CAL120 (average CI = 0.89) and HDQP1 (average CI = 0.65) (Figure 4B). CCT007093 was additive with paclitaxel in the two other paclitaxel-resistant cell lines SW527 and MT3 (average CI values not significantly different than 1 (*P > 0.05*)). Mithramycin was synergistic with paclitaxel in the two paclitaxel-sensitive lines MDA-MB-468 and MDA-MB-231, average CI value of 0.66 and 0.54, respectively, and the paclitaxel-resistant cell line HDQP1 average CI value 0.87. However, mithramycin and paclitaxel were antagonistic, average CI values significantly > 1, in reducing cell viability at high effective drug doses (IC_50 _and IC_25_) in the paclitaxel-resistant lines CAL120, SW527 and MT3 (Figure 4B). Collectively these data indicate that novel drug combinations with paclitaxel can effectively reduce cell viability of select paclitaxel-sensitive and importantly, paclitaxel-resistant TNBC cell lines.

## Discussion

Our RNAi screen represents a directed approach to identifying breast cancer relevant, druggable targets to enhance drug sensitivity. The screen was validated by our finding that several of the positive hits are genes that are known targets of paclitaxel sensitivity and have been clinically targeted in combination with taxanes [54,58,61,68,69]. We identified additional novel gene targets and respective agents that were not previously identified by drug sensitivity RNAi screens or whose inhibitors were not previously combined with paclitaxel.

We found PPM1D as a target for paclitaxel sensitivity in our RNAi screens and in follow-up studies observed synergistic inhibition of tumor cell growth with use of the PPM1D inhibitor CCT007093 in high PPM1D, wild-type p53 expressing MCF-7 cells. The oncogenic activity of PPM1D expression is attributed to its phosphatase activity and ability to deregulate tumor suppressor genes such as p53, Chk1, and p38 [71]. PPM1D contributes to the development of human cancers by suppressing p53 activation and thus has been an attractive therapeutic target in tumors that overexpress PPM1D and those with wild-type functional p53 activity [73]. Indeed, others have found that suppression of PPM1D expression by RNAi inhibits proliferation and induces apoptosis in breast cancer cell lines with wild-type p53 (BT-474) and those with PPM1D amplification (MCF-7 and ZR-75-1) [86]. However, the effect of inhibition of PPM1D on tumor cell growth and drug sensitivity is not limited to tumor cells that harbor these amplifications as we observed synergistic or additive activity of CCT007093 with paclitaxel in TNBC cell lines (MDA-MB-231 and MDA-MB-468, mutant p53 cell lines) including some paclitaxel-resistant lines. Likewise, Belova *et al. *identified chemical compounds that inhibit PPM1D activity and showed that these compounds could significantly inhibit tumor cell growth in MCF-7 cells and those with low PPM1D, mutant p53 expression MDA-MB-231 [74]. Interestingly, PPM1D inhibitors in both of these cell lines were able to potentiate the effects of doxorubicin but failed to enhance activity in other cell lines (MDA-MB-361) [74].

We found that mithramycin, an inhibitor of SP1 binding, could synergize with paclitaxel in some TNBC (basal-like) cell lines, MDA-MB-231, MDA-MB-468, and HDQP1. SP1 is a zinc finger transcription factor important in the regulation of genes involved in cell survival, growth and differentiation, and tumor development and progression [77]. SP1 cooperates with other prominent transcription factors including oncogenes such as MYC, which may contribute to tumor cell proliferation and growth [87,88]. MYC has recently been shown to have elevated activity and gene signatures present in basal-like TNBCs [89,90]. Thus, inhibiting SP1 binding with mithramycin may block oncogenic transcriptional activity and cooperate with anti-mitotic agents such as paclitaxel to inhibit tumor cell growth. In addition, SP1 is a potent transactivator of IGF-IR and EGFR, two prominent genes overexpressed in breast cancer cells (for example, MDA-MB-468) and both of which were identified as hits in our screen [91,92].

Despite extensive preclinical studies aimed at therapeutically targeting the TGFβ signaling pathway, there is a lack of reports in which TGFβ inhibitors are combined with paclitaxel. We found that the TGFβR inhibitor LY2109761 is synergistic with paclitaxel in breast cancer cells grown in 3D cultures but not 2D cultures, indicating the importance of performing drug combination in more than one growth context. TGFβ protects mammary epithelial cells from apoptosis in the absence of serum, which may be through activation of the PI3K/AKT cell survival pathway [80,93]. Thus, inhibition of TGFβ may sensitize cells that are grown in low-serum and/or anchorage-independent 3D conditions to apoptosis-inducing agents like paclitaxel. In support of this, others have shown that inhibition of TGFβ in human breast carcinoma cells grown in 3D cultures that secrete high levels of TGFβ increases the cytotoxic response to ionizing radiation and several chemotherapeutic drugs, namely cisplatin [94]. Likewise, inhibition of TGFβ can prevent radiation-induced acceleration of metastatic cancer progression [95,96]. On the contrary, Ahmed *et al. *showed that the loss of the ECM protein TGFβI is sufficient to induce specific resistance to paclitaxel and mitotic spindle abnormalities in ovarian cancer cells [97]. In ovarian and breast tumor specimens, TGFβI expression was shown to be tightly co-regulated with other genes that induce paclitaxel sensitivity, such as the adhesion glycoprotein, THBS1 [97].

The mechanism by which inhibition of TGFβ signaling cooperates with paclitaxel is not well understood. Intracellular TGFβ signaling proteins Smad2 and Smad3 bind microtubules, and upon TGFβ stimulation, these transcription factors dissociate from microtubules, are phosphorylated and relocate to the nucleus [98]. TGFβ signaling may serve as a growth promoter and/or enabling changes in tumor cell adhesion, migration, and host-tumor interactions [99]. Thus, loss of TGFβ signaling may sensitize cells to paclitaxel, an agent that can also alter adhesion and migration due to significant changes in microtubule dynamics that are required for these biological activities.

The ever-increasing volume of genomic information paired with bioinformatic and biostatistical analyses is making genotype-driven health care a reality. The tremendous amount of tumor-derived genomic information available now, and after completion of several large-scale cancer sequencing efforts, combined with biological validation of mutations to determine relevant drivers, will allow for much more facile identification of new targets for drug discovery, as well as more precise alignment of patients with a particular targeted therapy. Validation of putative drug targets through loss-of-function screening, similar to that performed herein, will likely be a frequently used approach to generate requisite pre-clinical data to investigate novel single agent and drug combinations. The exciting challenge ahead of us is to integrate the ever-expanding genomic information as quickly as possible for human benefit.

## Conclusions

We used a genomic-based approach that included loss-of-function RNAi screening to identify druggable targets involved in paclitaxel sensitivity in breast cancer cells. We identified pharmacological agents that target hits from our screens, several which sensitized breast cancer cells to paclitaxel. A potential translation of our discoveries is new treatment options for patients with TNBC disease, those without current clinically proven targeted therapies. In summary, we provide a platform in which integrated genomic information can be rationally used to design functional screens to identify druggable targets to improve current treatments or to discover novel cancer treatment strategies.

## Abbreviations

2D: two-dimensional; 3D: three-dimensional; CI: combination index; DMEM: Dulbecco's modified Eagle's medium; ECM: extracellular matrix; ER: estrogen receptor; IC: concentration that inhibits growth compared to control; NS: non-silencing; PR: progesterone receptor; RNAi: RNA interference; shRNA: short hairpin RNA; SI: sensitivity index; siRNA: small interfering RNA; TNBC: triple-negative breast cancer.

## Competing interests

The authors declare that they have no competing interests.

## Authors' contributions

JAB and JAP designed the study, analyzed data, and prepared the manuscript. CLA contributed to the interpretation of the results and discussion. CBM, BDL, and CP assisted with the RNAi screen and drug combination experiments. FY and YS provided a model and performed statistical analysis of the data generated from the RNAi screen. All authors read and approved the submitted manuscript.

## Supplementary Material

Additional file 1 **Growth conditions of breast cancer cell lines**. Cell culturing conditions for panel of triple-negative breast cancer cell lines. Click here for file

Additional file 2 **Knockdown of PPM1D expression correlates with increased paclitaxel sensitivity in breast cancer cells**. **A**. Non-silencing (NS) control, four individually designed PPM1D-1 (P1 to P4) or pooled PPM1D siRNAs (Dharmacon ON-TARGET plus) were transfected into MCF-7 and MDA-MB-468 cells. Cells were harvested 72 h after transfection, RNA purified, and the relative PPM1D mRNA expression was measured by quantitative real-time PCR. PPM1D mRNA knockdown by individual or pooled siRNAs is shown relative to non-silencing control. Error bars represent standard deviation from three independent experiments. **B**. Following transfection of siRNAs, as indicated above, MCF-7 and MDA-MB-468 cells were seeded in 12-well plates and treated with paclitaxel (0 to 3 nM) for two days. Cells were counted and quantified at 10 days after plating. The dose response curves of the surviving fractions are plotted relative to vehicle control treated cells. Error bars represent standard deviation of triplicate wells from three independent experiments. Click here for file

## References

[B1] Hortobagyi G Docetaxel in breast cancer and a rationale for combination therapy Oncology (Williston Park) 1997 11 11 15 9213321

[B2] Hortobagyi GN Paclitaxel-based combination chemotherapy for breast cancer Oncology (Williston Park) 1997 11 29 37 9110340

[B3] Ayers M Symmans WF Stec J Damokosh AI Clark E Hess K Lecocke M Metivier J Booser D Ibrahim N Valero V Royce M Arun B Whitman G Ross J Sneige N Hortobagyi GN Pusztai L Gene expression profiles predict complete pathologic response to neoadjuvant paclitaxel and fluorouracil, doxorubicin, and cyclophosphamide chemotherapy in breast cancer J Clin Oncol 2004 22 2284 2293 10.1200/JCO.2004.05.166 15136595

[B4] Dressman HK Hans C Bild A Olson JA Rosen E Marcom PK Liotcheva VB Jones EL Vujaskovic Z Marks J Dewhirst MW West M Nevins JR Blackwell K Gene expression profiles of multiple breast cancer phenotypes and response to neoadjuvant chemotherapy Clin Cancer Res 2006 12 819 826 10.1158/1078-0432.CCR-05-1447 16467094

[B5] Hess KR Anderson K Symmans WF Valero V Ibrahim N Mejia JA Booser D Theriault RL Buzdar AU Dempsey PJ Rouzier R Sneige N Ross JS Vidaurre T Gomez HL Hortobagyi GN Pusztai L Pharmacogenomic predictor of sensitivity to preoperative chemotherapy with paclitaxel and fluorouracil, doxorubicin, and cyclophosphamide in breast cancer J Clin Oncol 2006 24 4236 4244 10.1200/JCO.2006.05.6861 16896004

[B6] Thuerigen O Schneeweiss A Toedt G Warnat P Hahn M Kramer H Brors B Rudlowski C Benner A Schuetz F Tews B Eils R Sinn HP Sohn C Lichter P Gene expression signature predicting pathologic complete response with gemcitabine, epirubicin, and docetaxel in primary breast cancer J Clin Oncol 2006 24 1839 1845 10.1200/JCO.2005.04.7019 16622258

[B7] McGrogan BT Gilmartin B Carney DN McCann A Taxanes, microtubules and chemoresistant breast cancer Biochim Biophys Acta 2008 1785 96 132 18068131 10.1016/j.bbcan.2007.10.004

[B8] Noguchi S Predictive factors for response to docetaxel in human breast cancers Cancer Sci 2006 97 813 820 10.1111/j.1349-7006.2006.00265.x 16805818 PMC11158941

[B9] Villeneuve DJ Hembruff SL Veitch Z Cecchetto M Dew WA Parissenti AM cDNA microarray analysis of isogenic paclitaxel-and doxorubicin-resistant breast tumor cell lines reveals distinct drug-specific genetic signatures of resistance Breast Cancer Res Treat 2006 96 17 39 10.1007/s10549-005-9026-6 16322897

[B10] Burkhart CA Kavallaris M Band Horwitz S The role of beta-tubulin isotypes in resistance to antimitotic drugs Biochim Biophys Acta 2001 1471 O1 9 11342188 10.1016/s0304-419x(00)00022-6

[B11] Hasegawa S Miyoshi Y Egawa C Ishitobi M Tamaki Y Monden M Noguchi S Mutational analysis of the class I beta-tubulin gene in human breast cancer Int J Cancer 2002 101 46 51 10.1002/ijc.10575 12209587

[B12] Rouzier R Rajan R Wagner P Hess KR Gold DL Stec J Ayers M Ross JS Zhang P Buchholz TA Kuerer H Green M Arun B Hortobagyi GN Symmans WF Pusztai L Microtubule-associated protein tau: a marker of paclitaxel sensitivity in breast cancer Proc Natl Acad Sci USA 2005 102 8315 8320 10.1073/pnas.0408974102 15914550 PMC1149405

[B13] Estevez LG Cuevas JM Anton A Florian J Lopez-Vega JM Velasco A Lobo F Herrero A Fortes J Weekly docetaxel as neoadjuvant chemotherapy for stage II and III breast cancer: efficacy and correlation with biological markers in a phase II, multicenter study Clin Cancer Res 2003 9 686 692 12576436

[B14] Tham YL Gomez LF Mohsin S Gutierrez MC Weiss H Hilsenbeck SG Elledge RM Chamness GC Osborne CK Allred DC Chang JC Clinical response to neoadjuvant docetaxel predicts improved outcome in patients with large locally advanced breast cancers Breast Cancer Res Treat 2005 94 279 284 10.1007/s10549-005-9020-z 16261403

[B15] Learn PA Yeh IT McNutt M Chisholm GB Pollock BH Rousseau DL Jr Sharkey FE Cruz AB Kahlenberg MS HER-2/neu expression as a predictor of response to neoadjuvant docetaxel in patients with operable breast carcinoma Cancer 2005 103 2252 2260 10.1002/cncr.21037 15834928

[B16] Yu D Jing T Liu B Yao J Tan M McDonnell TJ Hung MC Overexpression of ErbB2 blocks Taxol-induced apoptosis by upregulation of p21Cip1, which inhibits p34Cdc2 kinase Mol Cell 1998 2 581 591 10.1016/S1097-2765(00)80157-4 9844631

[B17] Lafarge S Sylvain V Ferrara M Bignon YJ Inhibition of BRCA1 leads to increased chemoresistance to microtubule-interfering agents, an effect that involves the JNK pathway Oncogene 2001 20 6597 6606 10.1038/sj.onc.1204812 11641785

[B18] Zhou C Smith JL Liu J Role of BRCA1 in cellular resistance to paclitaxel and ionizing radiation in an ovarian cancer cell line carrying a defective BRCA1 Oncogene 2003 22 2396 2404 10.1038/sj.onc.1206319 12717416

[B19] Brooks TA Minderman H O'Loughlin KL Pera P Ojima I Baer MR Bernacki RJ Taxane-based reversal agents modulate drug resistance mediated by P-glycoprotein, multidrug resistance protein, and breast cancer resistance protein Mol Cancer Ther 2003 2 1195 1205 14617793

[B20] Mechetner E Kyshtoobayeva A Zonis S Kim H Stroup R Garcia R Parker RJ Fruehauf JP Levels of multidrug resistance (MDR1) P-glycoprotein expression by human breast cancer correlate with in vitro resistance to taxol and doxorubicin Clin Cancer Res 1998 4 389 398 9516927

[B21] Shabbits JA Mayer LD P-glycoprotein modulates ceramide-mediated sensitivity of human breast cancer cells to tubulin-binding anticancer drugs Mol Cancer Ther 2002 1 205 213 12467215

[B22] Chang JC Wooten EC Tsimelzon A Hilsenbeck SG Gutierrez MC Elledge R Mohsin S Osborne CK Chamness GC Allred DC O'Connell P Gene expression profiling for the prediction of therapeutic response to docetaxel in patients with breast cancer Lancet 2003 362 362 369 10.1016/S0140-6736(03)14023-8 12907009

[B23] Iwao-Koizumi K Matoba R Ueno N Kim SJ Ando A Miyoshi Y Maeda E Noguchi S Kato K Prediction of docetaxel response in human breast cancer by gene expression profiling J Clin Oncol 2005 23 422 431 10.1200/JCO.2005.09.078 15659489

[B24] Bauer JA Chakravarthy AB Rosenbluth JM Mi D Seeley EH De Matos Granja-Ingram N Olivares MG Kelley MC Mayer IA Meszoely IM Means-Powell JA Johnson KN Tsai CJ Ayers GD Sanders ME Schneider RJ Formenti SC Caprioli RM Pietenpol JA Identification of markers of taxane sensitivity using proteomic and genomic analyses of breast tumors from patients receiving neoadjuvant paclitaxel and radiation Clin Cancer Res 16 681 690 10.1158/1078-0432.CCR-09-1091 20068102 PMC2892225

[B25] Juul N Szallasi Z Eklund AC Li Q Burrell RA Gerlinger M Valero V Andreopoulou E Esteva FJ Symmans WF Desmedt C Haibe-Kains B Sotiriou C Pusztai L Swanton C Assessment of an RNA interference screen-derived mitotic and ceramide pathway metagene as a predictor of response to neoadjuvant paclitaxel for primary triple-negative breast cancer: a retrospective analysis of five clinical trials Lancet Oncol 2010 11 358 65 10.1016/S1470-2045(10)70018-8 20189874

[B26] Schwartz J Current combination chemotherapy regimens for metastatic breast cancer Am J Health Syst Pharm 2009 66 S3 8 10.2146/ajhp090438 19923317

[B27] Bartz SR Zhang Z Burchard J Imakura M Martin M Palmieri A Needham R Guo J Gordon M Chung N Warrener P Jackson AL Carleton M Oatley M Locco L Santini F Smith T Kunapuli P Ferrer M Strulovici B Friend SH Linsley PS Small interfering RNA screens reveal enhanced cisplatin cytotoxicity in tumor cells having both BRCA network and TP53 disruptions Mol Cell Biol 2006 26 9377 9386 10.1128/MCB.01229-06 17000754 PMC1698535

[B28] Honma K Iwao-Koizumi K Takeshita F Yamamoto Y Yoshida T Nishio K Nagahara S Kato K Ochiya T RPN2 gene confers docetaxel resistance in breast cancer Nat Med 2008 14 939 948 10.1038/nm.1858 18724378

[B29] Ji D Deeds SL Weinstein EJ A screen of shRNAs targeting tumor suppressor genes to identify factors involved in A549 paclitaxel sensitivity Oncol Rep 2007 18 1499 1505 17982636

[B30] MacKeigan JP Murphy LO Blenis J Sensitized RNAi screen of human kinases and phosphatases identifies new regulators of apoptosis and chemoresistance Nat Cell Biol 2005 7 591 600 10.1038/ncb1258 15864305

[B31] Menendez JA Vellon L Colomer R Lupu R Pharmacological and small interference RNA-mediated inhibition of breast cancer-associated fatty acid synthase (oncogenic antigen-519) synergistically enhances Taxol (paclitaxel)-induced cytotoxicity Int J Cancer 2005 115 19 35 10.1002/ijc.20754 15657900

[B32] Swanton C Marani M Pardo O Warne PH Kelly G Sahai E Elustondo F Chang J Temple J Ahmed AA Brenton JD Downward J Nicke B Regulators of mitotic arrest and ceramide metabolism are determinants of sensitivity to paclitaxel and other chemotherapeutic drugs Cancer Cell 2007 11 498 512 10.1016/j.ccr.2007.04.011 17560332

[B33] Whitehurst AW Bodemann BO Cardenas J Ferguson D Girard L Peyton M Minna JD Michnoff C Hao W Roth MG Xie XJ White MA Synthetic lethal screen identification of chemosensitizer loci in cancer cells Nature 2007 446 815 819 10.1038/nature05697 17429401

[B34] Adelaide J Finetti P Bekhouche I Repellini L Geneix J Sircoulomb F Charafe-Jauffret E Cervera N Desplans J Parzy D Schoenmakers E Viens P Jacquemier J Birnbaum D Bertucci F Chaffanet M Integrated profiling of basal and luminal breast cancers Cancer Res 2007 67 11565 11575 10.1158/0008-5472.CAN-07-2536 18089785

[B35] Bergamaschi A Kim YH Wang P Sorlie T Hernandez-Boussard T Lonning PE Tibshirani R Borresen-Dale AL Pollack JR Distinct patterns of DNA copy number alteration are associated with different clinicopathological features and gene-expression subtypes of breast cancer Genes Chromosomes Cancer 2006 45 1033 1040 10.1002/gcc.20366 16897746

[B36] Han W Jung EM Cho J Lee JW Hwang KT Yang SJ Kang JJ Bae JY Jeon YK Park IA Nicolau M Jeffrey SS Noh DY DNA copy number alterations and expression of relevant genes in triple-negative breast cancer Genes Chromosomes Cancer 2008 47 490 499 10.1002/gcc.20550 18314908

[B37] Neve RM Chin K Fridlyand J Yeh J Baehner FL Fevr T Clark L Bayani N Coppe JP Tong F Speed T Spellman PT DeVries S Lapuk A Wang NJ Kuo WL Stilwell JL Pinkel D Albertson DG Waldman FM McCormick F Dickson RB Johnson MD Lippman M Ethier S Gazdar A Gray JW A collection of breast cancer cell lines for the study of functionally distinct cancer subtypes Cancer Cell 2006 10 515 527 10.1016/j.ccr.2006.10.008 17157791 PMC2730521

[B38] Chin K DeVries S Fridlyand J Spellman PT Roydasgupta R Kuo WL Lapuk A Neve RM Qian Z Ryder T Chen F Feiler H Tokuyasu T Kingsley C Dairkee S Meng Z Chew K Pinkel D Jain A Ljung BM Esserman L Albertson DG Waldman FM Gray JW Genomic and transcriptional aberrations linked to breast cancer pathophysiologies Cancer Cell 2006 10 529 541 10.1016/j.ccr.2006.10.009 17157792

[B39] Leary RJ Lin JC Cummins J Boca S Wood LD Parsons DW Jones S Sjoblom T Park BH Parsons R Willis J Dawson D Willson JK Nikolskaya T Nikolsky Y Kopelovich L Papadopoulos N Pennacchio LA Wang TL Markowitz SD Parmigiani G Kinzler KW Vogelstein B Velculescu VE Integrated analysis of homozygous deletions, focal amplifications, and sequence alterations in breast and colorectal cancers Proc Natl Acad Sci USA 2008 105 16224 16229 10.1073/pnas.0808041105 18852474 PMC2571022

[B40] Shah SP Morin RD Khattra J Prentice L Pugh T Burleigh A Delaney A Gelmon K Guliany R Senz J Steidl C Holt RA Jones S Sun M Leung G Moore R Severson T Taylor GA Teschendorff AE Tse K Turashvili G Varhol R Warren RL Watson P Zhao Y Caldas C Huntsman D Hirst M Marra MA Aparicio S Mutational evolution in a lobular breast tumour profiled at single nucleotide resolution Nature 2009 461 809 813 10.1038/nature08489 19812674

[B41] Stephens PJ McBride DJ Lin ML Varela I Pleasance ED Simpson JT Stebbings LA Leroy C Edkins S Mudie LJ Greenman CD Jia M Latimer C Teague JW Lau KW Burton J Quail MA Swerdlow H Churcher C Natrajan R Sieuwerts AM Martens JW Silver DP Langerod A Russnes HE Foekens JA Reis-Filho JS van 't Veer L Richardson AL Borresen-Dale AL Complex landscapes of somatic rearrangement in human breast cancer genomes Nature 2009 462 1005 1010 10.1038/nature08645 20033038 PMC3398135

[B42] Nikolsky Y Sviridov E Yao J Dosymbekov D Ustyansky V Kaznacheev V Dezso Z Mulvey L Macconaill LE Winckler W Serebryiskaya T Nikolskaya T Polyak K Genome-wide functional synergy between amplified and mutated genes in human breast cancer Cancer Res 2008 68 9532 9540 10.1158/0008-5472.CAN-08-3082 19010930

[B43] Sjoblom T Jones S Wood LD Parsons DW Lin J Barber TD Mandelker D Leary RJ Ptak J Silliman N Szabo S Buckhaults P Farrell C Meeh P Markowitz SD Willis J Dawson D Willson JK Gazdar AF Hartigan J Wu L Liu C Parmigiani G Park BH Bachman KE Papadopoulos N Vogelstein B Kinzler KW Velculescu VE The consensus coding sequences of human breast and colorectal cancers Science 2006 314 268 274 10.1126/science.1133427 16959974

[B44] Wood LD Parsons DW Jones S Lin J Sjoblom T Leary RJ Shen D Boca SM Barber T Ptak J Silliman N Szabo S Dezso Z Ustyanksky V Nikolskaya T Nikolsky Y Karchin R Wilson PA Kaminker JS Zhang Z Croshaw R Willis J Dawson D Shipitsin M Willson JK Sukumar S Polyak K Park BH Pethiyagoda CL Pant PV The genomic landscapes of human breast and colorectal cancers Science 2007 318 1108 1113 10.1126/science.1145720 17932254

[B45] Boyd ZS Wu QJ O'Brien C Spoerke J Savage H Fielder PJ Amler L Yan Y Lackner MR Proteomic analysis of breast cancer molecular subtypes and biomarkers of response to targeted kinase inhibitors using reverse-phase protein microarrays Mol Cancer Ther 2008 7 3695 3706 10.1158/1535-7163.MCT-08-0810 19056674

[B46] Hennessy BT Gonzalez-Angulo AM Stemke-Hale K Gilcrease MZ Krishnamurthy S Lee JS Fridlyand J Sahin A Agarwal R Joy C Liu W Stivers D Baggerly K Carey M Lluch A Monteagudo C He X Weigman V Fan C Palazzo J Hortobagyi GN Nolden LK Wang NJ Valero V Gray JW Perou CM Mills GB Characterization of a naturally occurring breast cancer subset enriched in epithelial-to-mesenchymal transition and stem cell characteristics Cancer Res 2009 69 4116 4124 10.1158/0008-5472.CAN-08-3441 19435916 PMC2737191

[B47] Ruike Y Imanaka Y Sato F Shimizu K Tsujimoto G Genome-wide analysis of aberrant methylation in human breast cancer cells using methyl-DNA immunoprecipitation combined with high-throughput sequencing BMC Genomics 11 137 10.1186/1471-2164-11-137 20181289 PMC2838848

[B48] Andrews J Kennette W Pilon J Hodgson A Tuck AB Chambers AF Rodenhiser DI Multi-platform whole-genome microarray analyses refine the epigenetic signature of breast cancer metastasis with gene expression and copy number PLoS One 5 e8665 10.1371/journal.pone.0008665 20084286 PMC2801616

[B49] Iorio MV Ferracin M Liu CG Veronese A Spizzo R Sabbioni S Magri E Pedriali M Fabbri M Campiglio M Menard S Palazzo JP Rosenberg A Musiani P Volinia S Nenci I Calin GA Querzoli P Negrini M Croce CM MicroRNA gene expression deregulation in human breast cancer Cancer Res 2005 65 7065 7070 10.1158/0008-5472.CAN-05-1783 16103053

[B50] Mattie MD Benz CC Bowers J Sensinger K Wong L Scott GK Fedele V Ginzinger D Getts R Haqq C Optimized high-throughput microRNA expression profiling provides novel biomarker assessment of clinical prostate and breast cancer biopsies Mol Cancer 2006 5 24 10.1186/1476-4598-5-24 16784538 PMC1563474

[B51] Debnath J Muthuswamy SK Brugge JS Morphogenesis and oncogenesis of MCF-10A mammary epithelial acini grown in three-dimensional basement membrane cultures Methods 2003 30 256 268 10.1016/S1046-2023(03)00032-X 12798140

[B52] Lee GY Kenny PA Lee EH Bissell MJ Three-dimensional culture models of normal and malignant breast epithelial cells Nat Methods 2007 4 359 365 10.1038/nmeth1015 17396127 PMC2933182

[B53] Chou TC Talalay P Quantitative analysis of dose-effect relationships: the combined effects of multiple drugs or enzyme inhibitors Adv Enzyme Regul 1984 22 27 55 10.1016/0065-2571(84)90007-4 6382953

[B54] Modi S D'Andrea G Norton L Yao TJ Caravelli J Rosen PP Hudis C Seidman AD A phase I study of cetuximab/paclitaxel in patients with advanced-stage breast cancer Clin Breast Cancer 2006 7 270 277 10.3816/CBC.2006.n.040 16942645

[B55] Finn RS Press MF Dering J Arbushites M Koehler M Oliva C Williams LS Di Leo A Estrogen receptor, progesterone receptor, human epidermal growth factor receptor 2 (HER2), and epidermal growth factor receptor expression and benefit from lapatinib in a randomized trial of paclitaxel with lapatinib or placebo as first-line treatment in HER2-negative or unknown metastatic breast cancer J Clin Oncol 2009 27 3908 3915 10.1200/JCO.2008.18.1925 19620495 PMC2799151

[B56] Di Leo A Gomez HL Aziz Z Zvirbule Z Bines J Arbushites MC Guerrera SF Koehler M Oliva C Stein SH Williams LS Dering J Finn RS Press MF Phase III, double-blind, randomized study comparing lapatinib plus paclitaxel with placebo plus paclitaxel as first-line treatment for metastatic breast cancer J Clin Oncol 2008 26 5544 5552 10.1200/JCO.2008.16.2578 18955454 PMC2651098

[B57] Dai CL Tiwari AK Wu CP Su XD Wang SR Liu DG Ashby CR Jr Huang Y Robey RW Liang YJ Chen LM Shi CJ Ambudkar SV Chen ZS Fu LW Lapatinib (Tykerb, GW572016) reverses multidrug resistance in cancer cells by inhibiting the activity of ATP-binding cassette subfamily B member 1 and G member 2 Cancer Res 2008 68 7905 7914 10.1158/0008-5472.CAN-08-0499 18829547 PMC2652245

[B58] Campone M Levy V Bourbouloux E Berton Rigaud D Bootle D Dutreix C Zoellner U Shand N Calvo F Raymond E Safety and pharmacokinetics of paclitaxel and the oral mTOR inhibitor everolimus in advanced solid tumours Br J Cancer 2009 100 315 321 10.1038/sj.bjc.6604851 19127256 PMC2634724

[B59] Mondesire WH Jian W Zhang H Ensor J Hung MC Mills GB Meric-Bernstam F Targeting mammalian target of rapamycin synergistically enhances chemotherapy-induced cytotoxicity in breast cancer cells Clin Cancer Res 2004 10 7031 7042 10.1158/1078-0432.CCR-04-0361 15501983

[B60] Dai Q Ling YH Lia M Zou YY Kroog G Iwata KK Perez-Soler R Enhanced sensitivity to the HER1/epidermal growth factor receptor tyrosine kinase inhibitor erlotinib hydrochloride in chemotherapy-resistant tumor cell lines Clin Cancer Res 2005 11 1572 1578 10.1158/1078-0432.CCR-04-0993 15746062

[B61] Fountzilas G Pectasides D Kalogera-Fountzila A Skarlos D Kalofonos HP Papadimitriou C Bafaloukos D Lambropoulos S Papadopoulos S Kourea H Markopoulos C Linardou H Mavroudis D Briasoulis E Pavlidis N Razis E Kosmidis P Gogas H Paclitaxel and carboplatin as first-line chemotherapy combined with gefitinib (IRESSA) in patients with advanced breast cancer: a phase I/II study conducted by the Hellenic Cooperative Oncology Group Breast Cancer Res Treat 2005 92 1 9 10.1007/s10549-005-0322-y 15980985

[B62] Schafer-Hales K Iaconelli J Snyder JP Prussia A Nettles JH El-Naggar A Khuri FR Giannakakou P Marcus AI Farnesyl transferase inhibitors impair chromosomal maintenance in cell lines and human tumors by compromising CENP-E and CENP-F function Mol Cancer Ther 2007 6 1317 1328 10.1158/1535-7163.MCT-06-0703 17431110

[B63] Shoemaker AR Oleksijew A Bauch J Belli BA Borre T Bruncko M Deckwirth T Frost DJ Jarvis K Joseph MK Marsh K McClellan W Nellans H Ng S Nimmer P O'Connor JM Oltersdorf T Qing W Shen W Stavropoulos J Tahir SK Wang B Warner R Zhang H Fesik SW Rosenberg SH Elmore SW A small-molecule inhibitor of Bcl-XL potentiates the activity of cytotoxic drugs in vitro and in vivo Cancer Res 2006 66 8731 8739 10.1158/0008-5472.CAN-06-0367 16951189

[B64] Kutuk O Letai A Alteration of the mitochondrial apoptotic pathway is key to acquired paclitaxel resistance and can be reversed by ABT-737 Cancer Res 2008 68 7985 7994 10.1158/0008-5472.CAN-08-1418 18829556 PMC2603173

[B65] Xu R Sato N Yanai K Akiyoshi T Nagai S Wada J Koga K Mibu R Nakamura M Katano M Enhancement of paclitaxel-induced apoptosis by inhibition of mitogen-activated protein kinase pathway in colon cancer cells Anticancer Res 2009 29 261 270 19331159

[B66] MacKeigan JP Collins TS Ting JP MEK inhibition enhances paclitaxel-induced tumor apoptosis J Biol Chem 2000 275 38953 38956 10.1074/jbc.C000684200 11038347

[B67] Mukohara T Shimada H Ogasawara N Wanikawa R Shimomura M Nakatsura T Ishii G Park JO Janne PA Saijo N Minami H Sensitivity of breast cancer cell lines to the novel insulin-like growth factor-1 receptor (IGF-1R) inhibitor NVP-AEW541 is dependent on the level of IRS-1 expression Cancer Lett 2009 282 14 24 10.1016/j.canlet.2009.02.056 19345478

[B68] Ready NE Lipton A Zhu Y Statkevich P Frank E Curtis D Bukowski RM Phase I study of the farnesyltransferase inhibitor lonafarnib with weekly paclitaxel in patients with solid tumors Clin Cancer Res 2007 13 576 583 10.1158/1078-0432.CCR-06-1262 17255280

[B69] Khuri FR Glisson BS Kim ES Statkevich P Thall PF Meyers ML Herbst RS Munden RF Tendler C Zhu Y Bangert S Thompson E Lu C Wang XM Shin DM Kies MS Papadimitrakopoulou V Fossella FV Kirschmeier P Bishop WR Hong WK Phase I study of the farnesyltransferase inhibitor lonafarnib with paclitaxel in solid tumors Clin Cancer Res 2004 10 2968 2976 10.1158/1078-0432.CCR-03-0412 15131032

[B70] Hu L Hofmann J Lu Y Mills GB Jaffe RB Inhibition of phosphatidylinositol 3'-kinase increases efficacy of paclitaxel in in vitro and in vivo ovarian cancer models Cancer Res 2002 62 1087 1092 11861387

[B71] Lu X Nguyen TA Moon SH Darlington Y Sommer M Donehower LA The type 2C phosphatase Wip1: an oncogenic regulator of tumor suppressor and DNA damage response pathways Cancer Metastasis Rev 2008 27 123 135 10.1007/s10555-008-9127-x 18265945 PMC2362138

[B72] Rauta J Alarmo EL Kauraniemi P Karhu R Kuukasjarvi T Kallioniemi A The serine-threonine protein phosphatase PPM1 D is frequently activated through amplification in aggressive primary breast tumours Breast Cancer Res Treat 2006 95 257 263 10.1007/s10549-005-9017-7 16254685

[B73] Bulavin DV Demidov ON Saito S Kauraniemi P Phillips C Amundson SA Ambrosino C Sauter G Nebreda AR Anderson CW Kallioniemi A Fornace AJ Jr Appella E Amplification of PPM1 D in human tumors abrogates p53 tumor-suppressor activity Nat Genet 2002 31 210 215 10.1038/ng894 12021785

[B74] Belova GI Demidov ON Fornace AJ Jr Bulavin DV Chemical inhibition of Wip1 phosphatase contributes to suppression of tumorigenesis Cancer Biol Ther 2005 4 1154 1158 16258255 10.4161/cbt.4.10.2204

[B75] Rayter S Elliott R Travers J Rowlands MG Richardson TB Boxall K Jones K Linardopoulos S Workman P Aherne W Lord CJ Ashworth A A chemical inhibitor of PPM1 D that selectively kills cells overexpressing PPM1D Oncogene 2008 27 1036 1044 10.1038/sj.onc.1210729 17700519

[B76] Zannetti A Del Vecchio S Carriero MV Fonti R Franco P Botti G D'Aiuto G Stoppelli MP Salvatore M Coordinate up-regulation of Sp1 DNA-binding activity and urokinase receptor expression in breast carcinoma Cancer Res 2000 60 1546 1551 10749121

[B77] Black AR Black JD Azizkhan-Clifford J Sp1 and kruppel-like factor family of transcription factors in cell growth regulation and cancer J Cell Physiol 2001 188 143 160 10.1002/jcp.1111 11424081

[B78] Miller DM Polansky DA Thomas SD Ray R Campbell VW Sanchez J Koller CA Mithramycin selectively inhibits transcription of G-C containing DNA Am J Med Sci 1987 294 388 394 10.1097/00000441-198711000-00015 2962490

[B79] Tan AR Alexe G Reiss M Transforming growth factor-beta signaling: emerging stem cell target in metastatic breast cancer? Breast Cancer Res Treat 2009 115 453 495 10.1007/s10549-008-0184-1 18841463 PMC2693232

[B80] Muraoka-Cook RS Shin I Yi JY Easterly E Barcellos-Hoff MH Yingling JM Zent R Arteaga CL Activated type I TGFbeta receptor kinase enhances the survival of mammary epithelial cells and accelerates tumor progression Oncogene 2006 25 3408 3423 10.1038/sj.onc.1208964 16186809

[B81] Melisi D Ishiyama S Sclabas GM Fleming JB Xia Q Tortora G Abbruzzese JL Chiao PJ LY2109761, a novel transforming growth factor beta receptor type I and type II dual inhibitor, as a therapeutic approach to suppressing pancreatic cancer metastasis Mol Cancer Ther 2008 7 829 840 10.1158/1535-7163.MCT-07-0337 18413796 PMC3088432

[B82] Fransvea E Angelotti U Antonaci S Giannelli G Blocking transforming growth factor-beta up-regulates E-cadherin and reduces migration and invasion of hepatocellular carcinoma cells Hepatology 2008 47 1557 1566 10.1002/hep.22201 18318443

[B83] Liedtke C Mazouni C Hess KR Andre F Tordai A Mejia JA Symmans WF Gonzalez-Angulo AM Hennessy B Green M Cristofanilli M Hortobagyi GN Pusztai L Response to neoadjuvant therapy and long-term survival in patients with triple-negative breast cancer J Clin Oncol 2008 26 1275 1281 10.1200/JCO.2007.14.4147 18250347

[B84] Dent R Trudeau M Pritchard KI Hanna WM Kahn HK Sawka CA Lickley LA Rawlinson E Sun P Narod SA Triple-negative breast cancer: clinical features and patterns of recurrence Clin Cancer Res 2007 13 4429 4434 10.1158/1078-0432.CCR-06-3045 17671126

[B85] Anders CK Carey LA Biology, metastatic patterns, and treatment of patients with triple-negative breast cancer Clin Breast Cancer 2009 9 Suppl 2 S73 81 10.3816/CBC.2009.s.008 19596646 PMC2919761

[B86] Parssinen J Alarmo EL Karhu R Kallioniemi A PPM1 D silencing by RNA interference inhibits proliferation and induces apoptosis in breast cancer cell lines with wild-type p53 Cancer Genet Cytogenet 2008 182 33 39 10.1016/j.cancergencyto.2007.12.013 18328948

[B87] Parisi F Wirapati P Naef F Identifying synergistic regulation involving c-Myc and sp1 in human tissues Nucleic Acids Res 2007 35 1098 1107 10.1093/nar/gkl1157 17264126 PMC1851645

[B88] Wang LG Ferrari AC Mithramycin targets Sp1 and the androgen receptor transcription level: potential therapeutic role in advanced prostate cancer Translational Oncogenomics 2007 2006 19 31 PMC3642134 23662037

[B89] Chandriani S Frengen E Cowling VH Pendergrass SA Perou CM Whitfield ML Cole MD A core MYC gene expression signature is prominent in basal-like breast cancer but only partially overlaps the core serum response PLoS One 2009 4 e6693 10.1371/journal.pone.0006693 19690609 PMC2723908

[B90] Alles MC Gardiner-Garden M Nott DJ Wang Y Foekens JA Sutherland RL Musgrove EA Ormandy CJ Meta-analysis and gene set enrichment relative to er status reveal elevated activity of MYC and E2F in the "basal" breast cancer subgroup PLoS One 2009 4 e4710 10.1371/journal.pone.0004710 19270750 PMC2650420

[B91] Maor S Yosepovich A Papa MZ Yarden RI Mayer D Friedman E Werner H Elevated insulin-like growth factor-I receptor (IGF-IR) levels in primary breast tumors associated with BRCA1 mutations Cancer Lett 2007 257 236 243 10.1016/j.canlet.2007.07.019 17766039

[B92] Wang L Guan X Zhang J Jia Z Wei D Li Q Yao J Xie K Targeted inhibition of Sp1-mediated transcription for antiangiogenic therapy of metastatic human gastric cancer in orthotopic nude mouse models Int J Oncol 2008 33 161 167 18575762

[B93] Shin I Bakin AV Rodeck U Brunet A Arteaga CL Transforming growth factor beta enhances epithelial cell survival via Akt-dependent regulation of FKHRL1 Mol Biol Cell 2001 12 3328 3339 11694570 10.1091/mbc.12.11.3328 PMC60258

[B94] Ohmori T Yang JL Price JO Arteaga CL Blockade of tumor cell transforming growth factor-betas enhances cell cycle progression and sensitizes human breast carcinoma cells to cytotoxic chemotherapy Exp Cell Res 1998 245 350 359 10.1006/excr.1998.4261 9851876

[B95] Biswas S Guix M Rinehart C Dugger TC Chytil A Moses HL Freeman ML Arteaga CL Inhibition of TGF-beta with neutralizing antibodies prevents radiation-induced acceleration of metastatic cancer progression J Clin Invest 2007 117 1305 1313 10.1172/JCI30740 17415413 PMC1838926

[B96] Teicher BA Holden SA Ara G Chen G Transforming growth factor-beta in in vivo resistance Cancer Chemother Pharmacol 1996 37 601 609 10.1007/s002800050435 8612316

[B97] Ahmed AA Mills AD Ibrahim AE Temple J Blenkiron C Vias M Massie CE Iyer NG McGeoch A Crawford R Nicke B Downward J Swanton C Bell SD Earl HM Laskey RA Caldas C Brenton JD The extracellular matrix protein TGFBI induces microtubule stabilization and sensitizes ovarian cancers to paclitaxel Cancer Cell 2007 12 514 527 10.1016/j.ccr.2007.11.014 18068629 PMC2148463

[B98] Dong C Li Z Alvarez R Jr Feng XH Goldschmidt-Clermont PJ Microtubule binding to Smads may regulate TGF beta activity Mol Cell 2000 5 27 34 10.1016/S1097-2765(00)80400-1 10678166

[B99] Yang EY Moses HL Transforming growth factor beta 1-induced changes in cell migration, proliferation, and angiogenesis in the chicken chorioallantoic membrane J Cell Biol 1990 111 731 741 10.1083/jcb.111.2.731 1696268 PMC2116177

