# Supplementary material for: RNA interference (RNAi) screening approach identifies agents that enhance paclitaxel activity in breast cancer cells
Source: Breast Cancer Res. 2010 Jun 24;12(3):R41. doi: 10.1186/bcr2595 (PMC2917036; doi:10.1186/bcr2595)
Supplement: Additional file 1 — Growth conditions of breast cancer cell lines. Cell culturing conditions for panel of triple-negative breast cancer cell lines. [file bcr2595-S1.DOCX]

**Additional Table 1**

| **Cell Line** | **Isolation** | **Tumor Type** | **Source** | **Media** |
| --- | --- | --- | --- | --- |
| BT20 | PB | IDC | ATCC | EMEM 10%FBS |
| BT549 | PB | IDC | ATCC | RPMI 10% FBS |
| CAL120 | PE | AC | DSMZ | DMEM 10%FBS |
| CAL148 | PE | AC | DSMZ | DMEM 20% FBS + 2 mM L-glutamine + 1ug/mL EGF |
| CAL851 | PE | IGA | DSMZ | DMEM 10% FBS + 2 mM L-glutamine |
| HCC1143 | PB | IDC | ATCC | RPMI 10% FBS |
| HCC1395 | PB | DC | ATCC | RPMI 10% FBS |
| HCC1806 | PB | ASCC | ATCC | RPMI glutamax 10% FBS |
| HCC1937 | PB | DC | ATCC | RPMI glutamax 10% FBS |
| HCC70 | PB | DC | ATCC | RPMI 10% FBS |
| HDQP1 | PB | IDC | DSMZ | DMEM 10%FBS |
| HS578T | PB | CS | ATCC | DMEM 10%FBS |
| MDAMB157 | PB | MC | ATCC | DMEM 10%FBS |
| MDAMB231 | PE | IDC | ATCC | DMEM 10%FBS |
| MDAMB436 | PE | IDC | ATCC | DMEM 10%FBS |
| MDAMB453 | PF | AC | ATCC | DMEM 10%FBS |
| MDAMB468 | PE | DC | ATCC | DMEM 10%FBS |
| MFM223 | PE | AC | DSMZ | MEM (with Earle's salts) + 15% FBS + 2 mM L-glutamine + 1x insulin-transferrin-sodium selenite |
| MT3 | PE | C | DSMZ | RPMI 10% FBS |
| SUM149PT | PB | INF | Asterand | Hams F12 5%FBS +1mg/ml hydrocortisone +5mg/ml insulin |
| SUM159PT | PB | ANC | Asterand | Hams F12 5%FBS +1mg/ml hydrocortisone +5mg/ml insulin |
| SW527 | PE |  | ATCC | DMEM 10%FBS |
| Tumor Type (AC, adenocarcinoma; ANC, anaplastic carcinoma; ASCC, acantholytic squamous cell carcinoma; CS, Carcinosarcoma; DC, ductal carcinoma; C, carcinoma; CS, carcinosarcoma; IDC, invasive ductal carcinoma; IGA, invasive galactophoric adenocarinoma; INF, inflammatory ductual carcinoma; MC, metaplastic carcinoma)  Isolation (PB, primary breast; PE, pleural effusion; CN cutaneous nodule) | | | | |
