# Supplementary material for: RNA interference (RNAi) screening approach identifies agents that enhance paclitaxel activity in breast cancer cells
Source: Breast Cancer Res. 2010 Jun 24;12(3):R41. doi: 10.1186/bcr2595 (PMC2917036; doi:10.1186/bcr2595)
Supplement: Additional file 2 — Knockdown of PPM1D expression correlates with increased paclitaxel sensitivity in breast cancer cells. A. Non-silencing (NS) control, four individually designed PPM1D-1 (P1 to P4) or pooled PPM1D siRNAs (Dharmacon ON-TARGET plus) were transfected into MCF-7 and MDA-MB-468 cells. Cells were harvested 72 h after transfection, RNA purified, and the relative PPM1D mRNA expression was measured by quantitative real-time PCR. PPM1D mRNA knockdown by individual or pooled siRNAs is shown relative to non-silencing control. Error bars represent standard deviation from three independent experiments. B. Following transfection of siRNAs, as indicated above, MCF-7 and MDA-MB-468 cells were seeded in 12-well plates and treated with paclitaxel (0 to 3 nM) for two days. Cells were counted and quantified at 10 days after plating. The dose response curves of the surviving fractions are plotted relative to vehicle control treated cells. Error bars represent standard deviation of triplicate wells from three independent experiments. [file bcr2595-S2.PPT]

## Slide 1
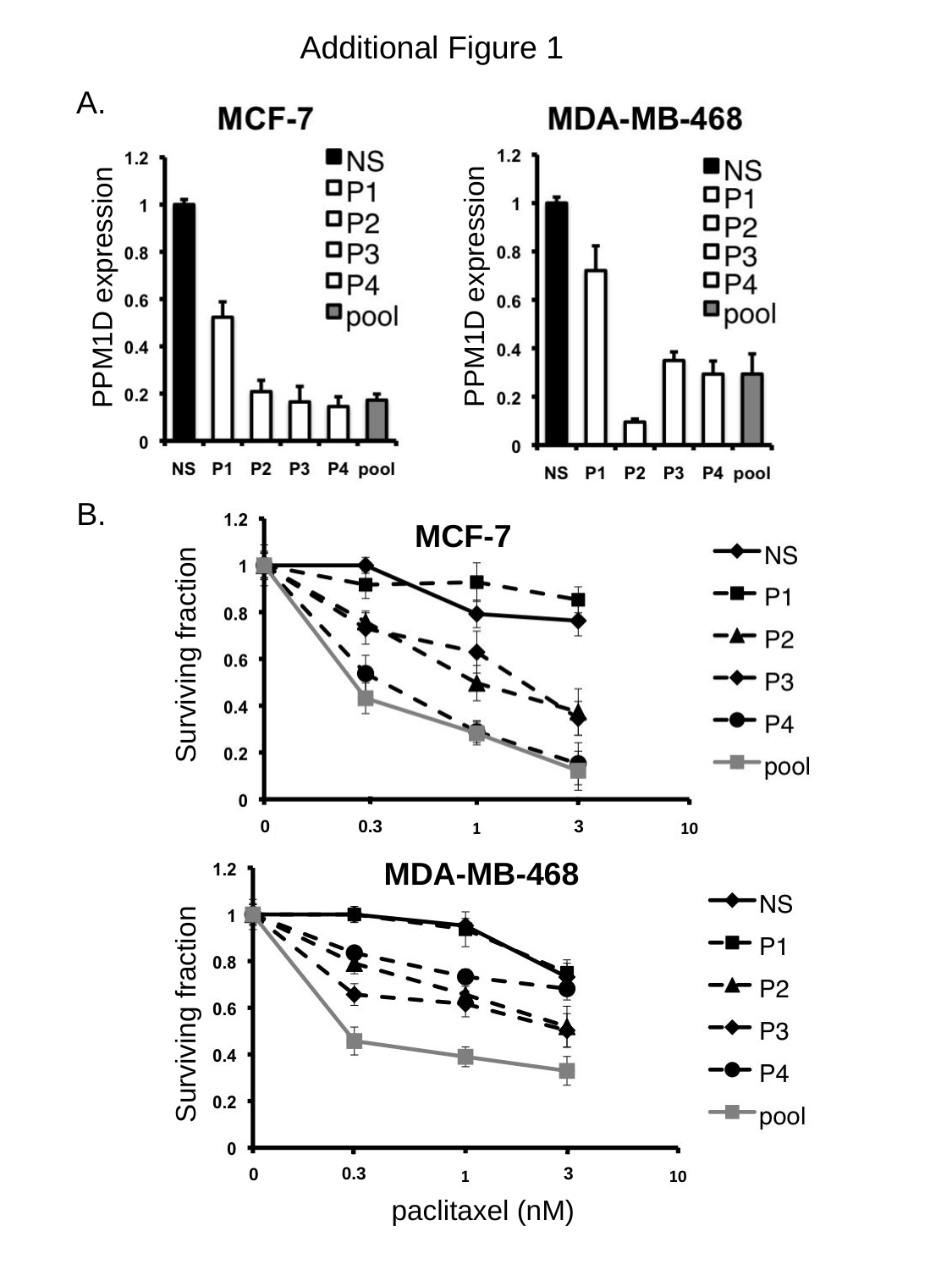

Additional Figure 1
A.
PPM1D expression
PPM1D expression
B.
MCF-7
Surviving fraction
0
MDA-MB-468
Surviving fraction
0
paclitaxel (nM)
0.3
3
0.3
3
